# Optimizing microgrid performance: Strategic integration of electric vehicle charging with renewable energy and storage systems for total operation cost and emissions minimization

**DOI:** 10.1371/journal.pone.0307810

**Published:** 2024-10-03

**Authors:** Obaid Aldosari, Ziad M. Ali, Shady H. E. Abdel Aleem, Mostafa H. Mostafa

**Affiliations:** 1 Department of Electrical Engineering, College of Engineering in Wadi Alddawasir, Prince Sattam Bin Abdulaziz University, Wadi Alddawasir, Saudi Arabia; 2 Electrical Engineering Department, Aswan Faculty of Engineering, Aswan University, Aswan, Egypt; 3 Department of Electrical Engineering, Institute of Aviation Engineering and Technology, Giza, Egypt; 4 Department of Electrical Power and Machines, International Academy of Engineering and Media Science, Giza, Egypt; Inner Mongolia University, CHINA

## Abstract

At present, renewable energy sources (RESs) and electric vehicles (EVs) are presented as viable solutions to reduce operation costs and lessen the negative environmental effects of microgrids (μGs). Thus, the rising demand for EV charging and storage systems coupled with the growing penetration of various RESs has generated new obstacles to the efficient operation and administration of these μGs. In this regard, this paper introduces a multi-objective optimization model for minimizing the total operation cost of the μG and its emissions, considering the effect of battery storage system (BSS) and EV charging station load. A day-ahead scheduling model is proposed for optimal energy management (EM) of the μG investigated, which comprises photovoltaics (PVs), fuel cells (FCs), wind turbines (WTs), BSSs, and EV charging stations, with shed light on the viability and benefits of connecting BSS with EV charging stations in the μG. Analyzing three case studies depending on the objective function—Case 1: execute EM to minimize total operation cost and maximize the profits of BSS, Case 2: execute EM to minimize total emission from the μG, and Case 3: execute EM to minimize total operation cost, maximize the profits of BSS, and minimize total emissions from the μG. The main aim of the presented optimization strategy is to achieve the best possible balance between reducing expenses and lessening the environmental impact of greenhouse gas emissions. The krill herd algorithm (KHA) is used to find the optimal solutions while considering various nonlinear constraints. To demonstrate the validity and effectiveness of the proposed solution, the study utilizes the KHA and compares the obtained results with those achieved by other optimization methods. It was demonstrated that such integration significantly enhances the μG’s operational efficiency, reduces operating costs, and minimizes environmental impact. The findings underscore the viability of combining EV charging infrastructure with renewable energy to meet the increasing energy demand sustainably. The novelty of this work lies in its multi-objective optimization approach, the integration of EV charging and BSS in μGs, the comparison with other optimization methods, and the emphasis on sustainability and addressing energy demand through the utilization of renewable energy and EVs.

## 1. Introduction

### 1.1 Background and motivation

The demand for energy has increased worldwide as modern societies’ population and energy use have dramatically increased. Total energy consumption is estimated to increase by 53% by 2035. Since the 2000s, there has been a growing awareness of greenhouse gas (GHG) emissions and environmental degradation caused by the substantial reliance on fossil fuels. At present, there are many international calls and strategies to face these issues [1–3], such as using renewable energy sources (RESs) as an alternative energy source. Unfortunately, the transportation and energy production sectors play a role in carbon dioxide (CO_2_) emissions, contributing to 64% of total emissions [4]. These emissions have resulted in consequences leading to widespread public concern [5]. Thus, integrating RESs with electric vehicles (EVs) is crucial to meet the necessary reduction goals for CO_2_ emissions in both the transportation and power sectors [6]. Additionally, the transportation industry has become a significant energy consumer, significantly impacting the need for electrical power sources [7].

Extensive research has been conducted to study the distinctive load characteristics of electrical power systems. EVs, a new type of electrical load/supply, are gaining recognition for their potential expansion. Plug-in electric vehicles (PEVs) are becoming more popular due to their reduced carbon emissions and the presence of government incentives [8]. Renewable energy sources like photovoltaics (PVs) and wind turbines (WTs) are often combined with EVs and battery storage systems (BSSs) in hybrid power plants, energy hubs, and microgrids (μGs) [9]. BSS plays a crucial role in the efficient operation and administration of μGs [10]. BSS enables load shifting by storing excess energy during periods of low demand and supplying it during peak demand periods. By optimizing the use of stored energy, BSS helps reduce peak demand from the grid, which can be costly [11]. This load management capability reduces the need for additional generation capacity and grid infrastructure upgrades, resulting in cost savings. BSS facilitates the integration of intermittent RESs, such as solar and wind, into microgrids. It stores surplus energy generated during periods of high renewable energy production and releases it when there is insufficient generation. This smoothes out the variability of renewable energy, enhances grid stability, and reduces reliance on fossil fuel-based backup generation, leading to lower operational costs and reduced emissions. BSS can provide ancillary services to the microgrid and the main grid, enhancing system reliability and stability [12]. BSS can respond rapidly to frequency fluctuations, voltage regulation, and grid imbalances, improving power quality and reducing the need for costly grid infrastructure upgrades. By providing these grid support services, BSS contributes to cost savings and facilitates the integration of renewable energy, ultimately reducing emissions. BSS allows microgrid operators to optimize energy usage based on time-of-use pricing. During periods of low electricity prices, BSS can store energy, which can be discharged during high-price periods, reducing the overall cost of electricity consumption. This optimization helps reduce electricity expenses and can incentivize the use of renewable energy sources during peak demand periods, further reducing emissions [13].

**Fig 1:**
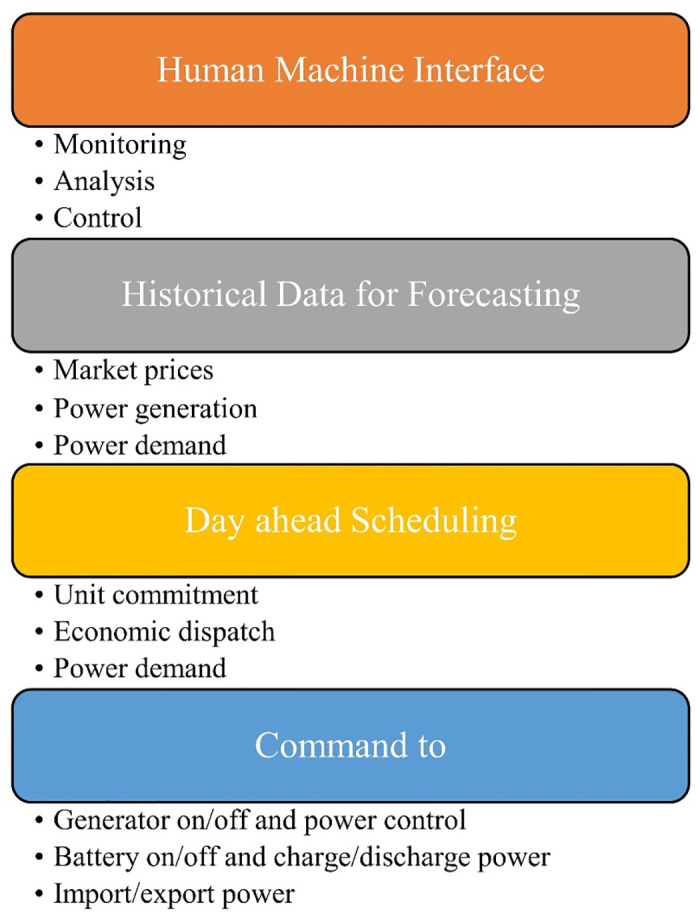
Energy management hierarchical system.

The system’s efficiency and power generation costs are greatly impacted by the strategic use of batteries and resource management. Energy management systems (EMSs) are essential for regulating energy in a μG to match the load need [14]. With the rising adoption of RESs and the emergence of new technologies such as energy storage systems and μGs [15], the optimization of energy resource management has become more crucial. EMSs provide real-time monitoring and control of energy distribution, enabling the seamless integration of μGs into power grids [16]. An EMS is responsible for overseeing a μG’s component known as a μG central control (μGCC) unit to ensure safe, dependable, and cost-effective operation [17]. Fig 1 illustrates the necessity of employing a hierarchical control approach with multiple layers to represent the μG environment as a unified entity.

Also, Fig 1 shows that initially, the data for power demand, power generation, and market price is collected. EM is done to determine the output of each unit considering all operation constraints of each power generation and μG, and then this is implemented in reality [18, 19]. The integration of EV charging with RESs and storage systems is a concept that aims to maximize the benefits of clean energy generation while efficiently managing EV charging and grid interactions. By integrating EV charging with RESs like PV or WT, we can significantly reduce our reliance on fossil fuels for transportation and electricity generation. This integration allows us to tap into clean, RES, which in turn leads to a reduction in greenhouse gas emissions [20]. EVs produce zero tailpipe emissions, meaning they don’t release any harmful pollutants while driving. However, it’s important to consider the source of electricity used to charge these vehicles. If the electricity comes from fossil fuel-based power plants, the overall emissions reduction potential is limited. That’s where the integration with RESs becomes crucial. The widespread adoption of EVs can present challenges and negative impacts on the power network [21].

According to the global EV outlook 2024 report published by the International Energy Agency (IEA), the global new registrations of EVs reached nearly 14 million units in 2023. This substantial increase in EV adoption has led to the total number of EVs on the world’s roads surpassing 40 million vehicles. Therefore, EVs have become an essential load in the electrical network that must be considered. The simultaneous charging of multiple EVs, particularly during peak hours, can strain the power grid and require costly infrastructure upgrades. It can lead to grid congestion, voltage stability issues, and increased peak demand. Distribution network planning becomes crucial to handle the increased load and ensure reliable power distribution. Additionally, EV charging can introduce power quality issues and require careful management to address harmonics and power factor concerns [22]. A BSS is one of the promising facilities that can mitigate the challenges that result from the adoption of EVs in an MG. BSS may improve the hosting capacity of the EV charging station to increase the number of EVs that can charge from it simultaneously and boost MG’s dependability to raise its resilience to recover swiftly from disruptions caused by the station [12]. This motivated the authors to conduct research on the optimization of μG performance through the integration of EV charging with renewable energy and storage systems.

Thus, connecting BSS with EV charging stations in microgrids offers several benefits in terms of operational efficiency, cost reduction, and environmental impact [23]. BSS can help balance the load by absorbing excess energy during periods of low demand and supplying it to EV charging stations during peak demand. This load balancing optimizes the utilization of available energy resources, reduces strain on the grid, and improves the overall operational efficiency of the microgrid [10]. By using BSS to manage the charging of EVs, microgrids can mitigate grid congestion issues caused by multiple EVs charging simultaneously. BSS can distribute the charging load intelligently, considering grid constraints and available capacity, to prevent overloading and ensure a reliable power supply to both EVs and other critical loads [23]. BSS can help shave peak demand during charging periods, reducing demand charges imposed by utilities. By avoiding peak demand spikes, microgrids can significantly lower electricity costs associated with high-demand tariffs, thus reducing operational expenses [13]. BSS can store excess energy during low-cost periods and discharge it during high-cost periods. By leveraging time-of-use pricing, microgrids can optimize the charging of EVs to align with cheaper electricity rates, resulting in cost savings. BSS coupled with EV charging stations enables better integration of renewable energy sources into microgrids. Excess renewable energy generated during periods of high production can be stored in the BSS and subsequently used to charge EVs [20]. This reduces the reliance on fossil fuel-based energy sources and promotes the use of clean energy, thereby reducing greenhouse gas emissions and environmental impact. By promoting the adoption of electric vehicles and utilizing renewable energy sources, the overall emissions associated with transportation and energy consumption can be reduced [22]. As EVs replace conventional combustion engine vehicles, the direct emissions from transportation are eliminated, while using renewable energy for charging further reduces indirect emissions from the electricity generation process [20].

### 1.2 Literature overview

Nodehi et al. [24] investigated the effect of the optimal EM of distributed generations (DGs) and EVs on the total operation cost (*T*_*cost*_) and total emission (*T*_*em*_) of the μG, considering the demand response. Rezaei et al. [25] presented robust optimization to scheduling the energy of μG with the integration of RESs and EVs, considering the uncertainty of RESs to increase μG profits and reduce its emissions. Tan and Chen [26] presented an optimization method to obtain the economic scheduling of μG to minimize operation costs, power losses, and total emissions, considering the operation constraints of transmission lines. Mohamed et al. [27] suggested a stochastic fuzzy technique to get optimal energy management for an μG, assessing EVs and different types of RESs to minimize the total operation cost of the μG. Mohamed et al. [28] presented an optimal approach based on scenario generation to schedule the energy, considering the uncertainties of RESs on the power losses, cost function, and voltage of the system. The model maintains the proper precision for modeling uncertainty while requiring less computing work than Monte Carlo.

Sedighizadeh et al. [29] investigated the effects of responsive loads and the stochastic behavior of electric cars as demand-side management tools on the effective functioning of a grid-connected μG that combines power, heat, and cooling systems. Li et al. [30] presented an optimization algorithm to obtain optimal operation of the μG, including EVs and RESs, to minimize the total operation cost of the μG. Gholami et al. [31] introduced a new management strategy to schedule the output power of EVs and DG units to minimize the operation cost of the distribution system, considering the uncertainty of electrical parameters. Doosti et al. [32] presented a management strategy to obtain optimal scheduling of EVs, energy storage, and RESs, considering the uncertainty of RESs to save energy. Zhang and Chen [33] introduced an optimization approach to optimize the energy of EVs and investigate the effect of EV load on the main grid. Kurukuru et al. [34] presented a fuzzy interference system-based EM of a single μG; μG profit is maximized using a genetic algorithm without any consideration of how the suggested approach would be affected by renewable generation. Pirouzi and Aghaei [35] introduced a model to manage the energy of the smart distribution network based on cooperation between the distribution system operator (DSO) and EV parking lots to improve network voltage security. Momen et al. [36] introduced a two-stage stochastic approach to obtain the EM of the distribution system based on the load control and the master-slave control techniques considering the diesel generators and PV units with EVs.

Within the context of integrating traffic networks and power grids, Lixun et al. [37] developed a comprehensive evaluation system and methodology for EV charging networks. Initially, an EV travel model is constructed, utilizing a trip probability matrix to analyze the geographical and temporal characteristics of EV usage. Subsequently, the interconnectedness among users, the road network, the power grid, and the charging infrastructure is examined. The study proposes four critical criteria: user feedback, the operational impact on the road network, the functionality of the charging network, and the influence on the power grid’s operation. For each criterion, specific evaluation indices are established, culminating in a holistic evaluation index system.

Subramaniam and Singh [38] detailed a strategic optimization approach for the charging of EVs and the selection of optimal installation sites. The main objective is to develop a charging network that is cost-effective while maintaining the operational integrity of the distribution network. The methodology addresses these challenges by employing renewable energy sources and meta-heuristic algorithms for optimal planning, taking into consideration the impacts of various factors. Consequently, this study introduces a novel perspective on managing the distribution of RES and charging station challenges, advocating for a multi-objective approach that incorporates the characteristics of charging stations.

Wenchao et al. [39] proposed a methodology to ascertain the optimal number of charging stations alongside a pricing strategy for EVs, considering various configurations of component commonality. The study explored four distinct scenarios characterized by differing common components and levels of quality. For each scenario, the optimal quantity of charging stations and the corresponding EV pricing were determined. Subsequently, through numerical simulations, the researchers evaluated the most favorable solutions and manufacturer profits across these scenarios, yielding insightful managerial implications.

To meet the growing demand for EVs, Hao et al. [40] developed a two-stage optimization framework aimed at enhancing the capacity and efficiency of charging stations. This framework systematically identifies the optimal capacity for generating renewable energy and the most effective timing for electricity distribution. By analyzing EV arrival patterns and demand profiles with real-world data, they established a viable model for EV service requests. Numerical results illustrate the optimal configuration for an EV charging station powered by renewable sources, showcasing the best mix of wind and solar power generation alongside the optimal electricity distribution schedule. Ahmad et al. [41] investigated the economic advantages of employing EVs as a temporary BSS within an μG that incorporates PV units. The formulation of the problem considered various factors, including the feed-in tariff, incentives for EV owners, and the capital costs associated with charging stations. To optimize the profitability of the proposed system, a planning algorithm was developed to determine the requisite number of EV charging stations (EVCSs).

### 1.3 Contribution and organization

In this study, the optimal EM of an μG, encompassing PVs, FCs, WTs, ESSs, and EVCSs, is optimized using the innovative krill herd algorithm (KHA), an algorithm that has garnered significant attention. This paper delivers an optimization strategy for managing the energy of an μG to fulfill multi-objective functions, including the minimization of total operational costs, maximization of BSS profits, and reduction of total emissions. The novelty of this work can be summarized as follows:

1. Multi-objective Optimization: The manuscript presents a multi-objective optimization model that simultaneously considers the microgrid’s total operation cost and emissions. This approach allows for a comprehensive analysis and decision-making process that aims to achieve the best balance between reducing expenses and minimizing the environmental impact of greenhouse gas emissions.
2. Integration of EV Charging and BSS: The manuscript highlights the benefits of connecting BSS with EV charging stations in microgrids. By intelligently managing the charging load and utilizing stored energy during peak demand, the integration of EVs and BSSs optimizes the utilization of available energy resources, reduces strain on the grid, and improves the overall operational efficiency of the microgrid.
3. Comparison with Other Optimization Methods: The study utilizes the krill herd algorithm (KHA) as an optimization technique and compares the results obtained with those other optimization methods. This comparison demonstrates the validity and effectiveness of the proposed solution. It shows that integrating EV charging infrastructure with renewable energy significantly enhances the microgrid’s operational efficiency, reduces operating costs, and minimizes environmental impact.
4. Sustainability and Energy Demand: The manuscript emphasizes the importance of integrating RESs with EVs to sustainably meet the increasing energy demand. By tapping into clean energy sources and reducing reliance on fossil fuels, adopting EVs and utilizing renewable energy help reduce greenhouse gas emissions and address environmental concerns.

The main contributions of this research are outlined as follows:

- A day-ahead scheduling model for optimal EM is introduced for interconnected μGs, incorporating PVs, FCs, WTs, ESSs, and EVCSs.
- A day-ahead scheduling model is introduced to determine the optimal operation schedule for each distributed generator within the interconnected microgrids depending on the objective function, considering factors such as energy generation, energy demand, grid constraints, and economic considerations.
- The study examines three case studies that focus on different objective functions related to implementing Energy Management (EM) within the microgrid (μG).
- The first case study aims to lower the total operational costs of the μG and increase the profits of the BSS. The EM strategies and scheduling decisions are optimized to minimize costs associated with energy generation, storage, and consumption while maximizing the revenue generated by the BSS.
- The second case study focuses on reducing the total emissions from the μG. The EM strategies and scheduling decisions are optimized to minimize the carbon footprint of the μG by maximizing the utilization of renewable energy sources, optimizing energy dispatch, and minimizing reliance on fossil fuel-based generation.
- The third case study combines the objectives of the first two studies. It aims to implement EM strategies that simultaneously decrease the total operational costs, enhance the profits of the BSS, and reduce the total emissions from the μG. The optimization model considers the trade-offs and synergies between these objectives to find a balanced solution that provides economic benefits, environmental sustainability, and efficient energy management.
- The optimization strategy employed in this study aims to achieve an optimal balance between cost reduction and the mitigation of environmental impacts associated with greenhouse gas (GHG) emissions. By considering both economic and environmental objectives, the strategy seeks to identify solutions that minimize operational costs while simultaneously reducing the carbon footprint of the microgrid (μG).
- To demonstrate the validity and effectiveness of the proposed solution, the study utilizes the Krill Herd Algorithm (KHA) and compares the obtained results with those achieved by other optimization methods, namely the Firefly (FF), Cuckoo (CK), and Golden Section Search (GSS) algorithms. This comparative analysis aims to evaluate the performance and efficiency of the KHA in solving the optimization problem at hand.

The article organizes the study into key sections, following the introduction that sets the context by discussing the global increase in energy demand, the significance of RESs and EVs for reducing carbon emissions, and the challenges in managing μGs, and explores the literature on EM, and the models used for efficient μGs operation. Further, Section 2 presents a schematic representation of the system under investigation with the formulation and discussion of its various components, PVs, FCs, WTs, ESSs, and EVCSs. Section 3 presents the problem formulation, objectives and constraints, and the optimization algorithm used. The main contributions include a proposed day-ahead scheduling model for interconnected microgrids, the benefits of incorporating BSS with EV charging stations, and the analysis of case studies to evaluate the model’s effectiveness in optimizing operation costs, maximizing BSS profits, and minimizing emissions. In this regard, Section 4 presents the results obtained and the discussion. The paper concludes with a summary of findings and potential areas for future research in Section 5.

## 2. System configuration

**Fig 2:**
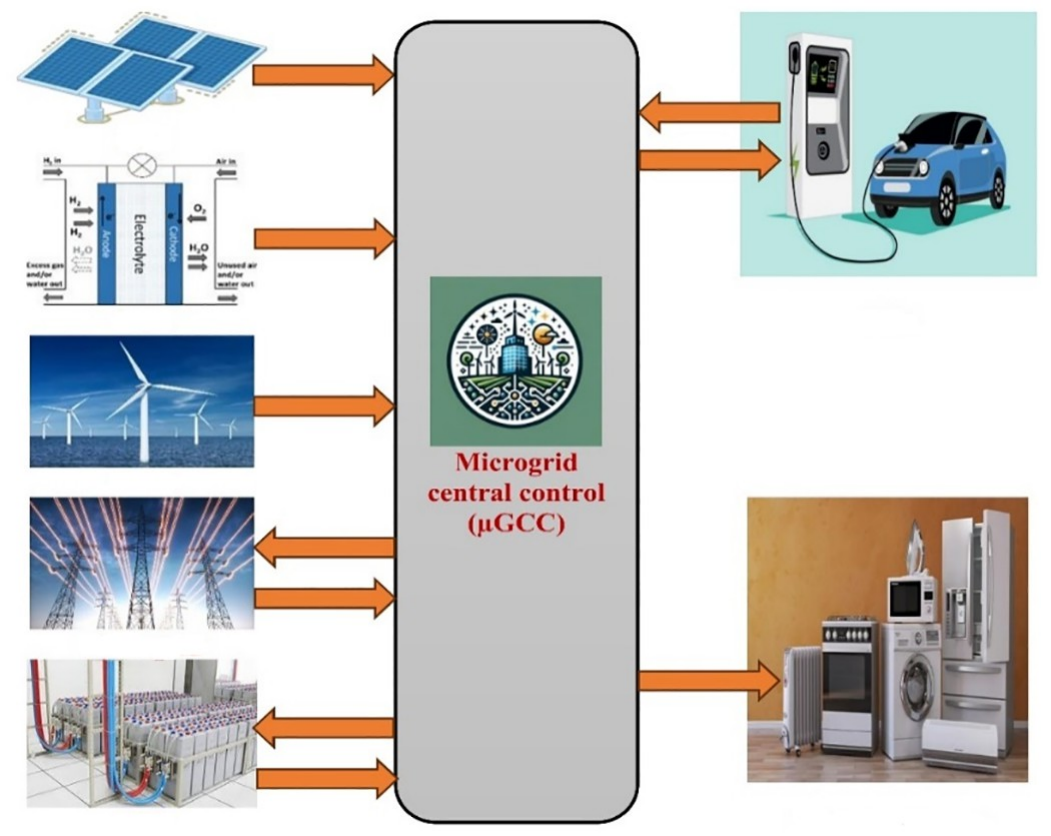
Energy management scheme’s structure.

Fig 2 illustrates the framework of the proposed energy management strategy. In this structure, the μG central control (μGCC) gathers energy pricing proposals from various generation units [42], taking into account the main grid’s energy market price and operational constraints of both the generation units and the μG itself. Furthermore, it incorporates the concept of vehicle-to-grid (V2G) energy transfer, leveraging the storage capacity of EVs [43]. The diagram also showcases the dynamic interactions within the modern grid model, highlighting bidirectional power flow between the EV and the power grid, as well as between the BSS and the power grid.

### 2.1 Photovoltaic (PV) units

The method of generating PV power involves converting solar radiation energy into electrical power through physical means. The solar cell is similar to a *pn* junction diode [44]. The output voltage is used to express the output current as follows:

$$P_{pv,t}=N^{PV}P_{rated}^{PV}\left(\frac{G}{G_{0}}\right)\left(1-T_{C}\left(T_{A}-25\right)\right)\eta^{inv}\eta^{rel}$$ (1)

where *P*_*pv*,*t*_ represents the output power of the PV units, and $P_{rated}^{PV}$ denotes the units’ rated power. *N*^*PV*^ indicates the total number of PV modules. *G* and *G*_0_ refer to the actual global solar irradiance and the standard test condition irradiance, measured in watts per square meter (W/m^2^), respectively. *T*_*A*_ stands for the ambient temperature, while *T*_*C*_ is the coefficient that describes how the PV’s maximum power output changes with temperature. *η*^*inv*^ and *η*^*rel*^ are the inverter’s efficiency and the PV modules’ relative efficiency, respectively [45].

### 2.2 Wind turbine (WT) units

Wind serves as the fundamental energy source for these turbines, as they convert wind energy into electrical power. The power generation capacity of these turbines is directly proportional to the speed of the wind [46].

$$P_{wt,t}=\left\{\begin{array}{cc} 0 & v_{t}^{WT}<v_{cut-in}^{WT}\\ P_{rated}^{WT}\left(\frac{{\left(v_{t}^{WT}\right)}^{3}-{\left(v_{cut-in}^{WT}\right)}^{3}}{{\left(v_{rated}^{WT}\right)}^{3}-{\left(v_{cut-in}^{WT}\right)}^{3}}\right) & v_{cut-in}^{WT}\leq v_{t}^{WT}<v_{rated}^{WT}\\ P_{rated}^{WT} & v_{rated}^{WT}\leq v_{t}^{WT}<v_{cut-out}^{WT}\\ 0 & v_{t}^{WT}\geq v_{cut-out}^{WT} \end{array}\right.$$ (2)

where, *P*_*wt*,*t*_ and $P_{rated}^{WT}$ denote the output power and the rated power of the WTs respectively. The terms $v_{t}^{WT}$ and $v_{rated}^{WT}$ correspond to the wind speed at the current time step and the WT’s rated wind speed, respectively. Moreover, $v_{cut-in}^{WT}$ and $v_{cut-out}^{WT}$ refer to the minimum and maximum wind speeds at which the WTs start and stop operating, respectively [47].

### 2.3 Fuel cells (FCs)

A fuel cell (FC) is an electrochemical apparatus that generates electrical power through the reaction of specific substances. One prevalent model, known as the polymer electrolyte membrane (PEM) fuel cell, PEMFC, harnesses a chemical process involving hydrogen and oxygen to produce electricity, with water as a byproduct [48]. This PEMFC is characterized by its semipermeable membrane, which facilitates the movement of protons but prevents electrons from passing through, thus distinguishing it as a proton-exchange membrane fuel cell due to its unique operation [49].

**Fig 3:**
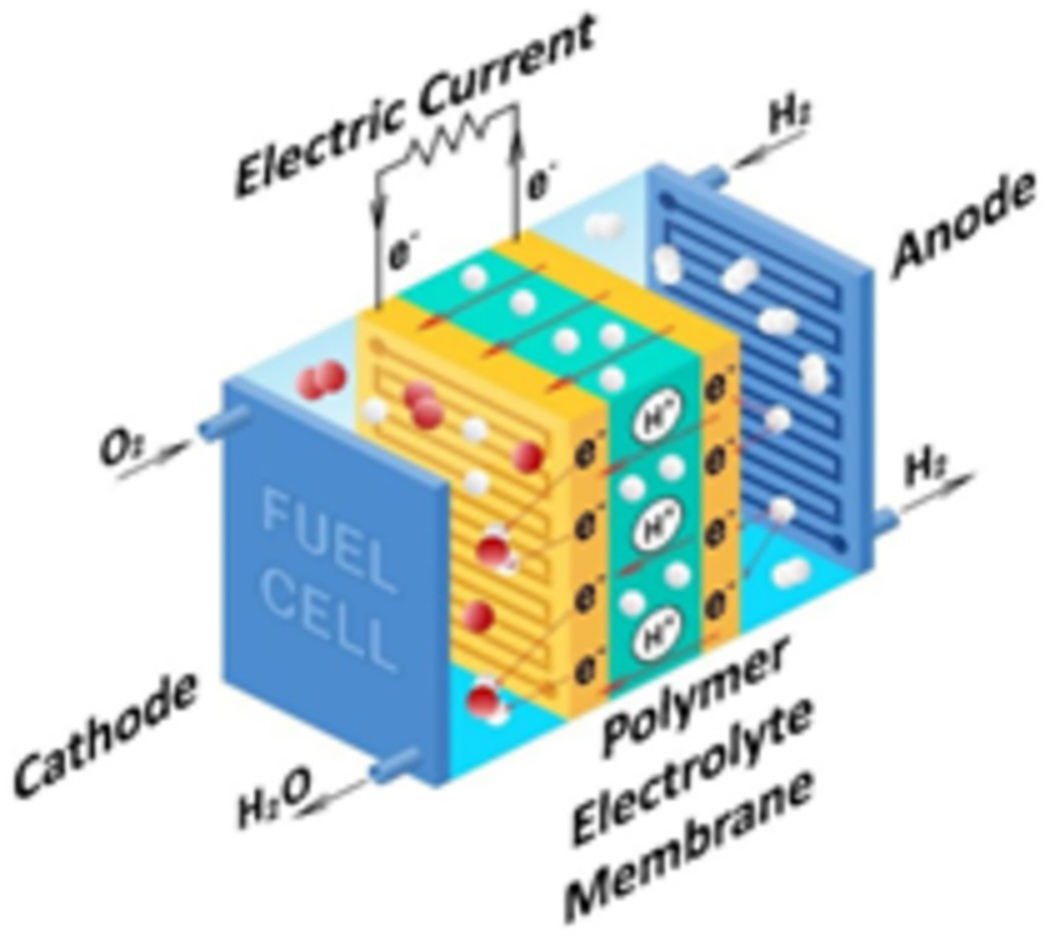
Configuration of PEMFC.

In the operation of the FC, hydrogen atoms are separated into protons and electrons upon arrival at the anode side. The electrons then take a path through an external circuit, generating electrical current, whereas the protons move directly through the membrane to the other side [50]. At the cathode end, the reunion of protons and electrons with incoming oxygen results in the production of heat and water, completing the chemical process. FCs, by bypassing the need for the combustion of traditional fuels, avoid the pollution associated with conventional fuel burning, thereby representing a sustainable and clean energy source. Fig 3 illustrates the fundamental principles of a PEMFC’s operation [51].

The Nernst equation relates the cell voltage (*E*) of the FC to the standard cell potential (*E*^0^), the gas constant (*R*), the temperature (*T*) in Kelvin, the Faraday constant (*F*), number of electrons transferred in the reaction (*n*) and the concentrations of reactants and products [52], where *Q* represents the reaction quotient (ratio of product concentrations to reactant concentrations).


$$E=E^{0}-\left(\frac{RT}{nF}\right).ln(Q)$$ (3)

The relationship between power (*P*_*fc*,*t*_) in watts, cell voltage in the FC (voltage across the FC) and *I* represents the electric current flowing through the FC can be described by the following equation [53] and:

$$P_{fc,t}=E.I$$ (4)

### 2.4 Battery storage system (BSS)

Rechargeable batteries, also known as energy storage devices, play a vital role in storing energy derived from both renewable and non-renewable sources for subsequent use [54]. These devices, functioning on direct current (DC), effectively bridge the gap between energy supply and demand by releasing stored electrochemical energy as required to meet electrical demands. BSS emerge as a pivotal technology for μGs in light of the increasing reliance on RESs and the global ambition to achieve net-zero carbon emissions. The deployment of BSS is instrumental in advancing toward net-zero energy goals, offering a critical pathway towards the adoption of green energy solutions [55].

### 2.5 Electric vehicles (EVs)

The charging and discharging patterns of EVs are influenced by several key factors, including the number of EVs connected for charging, the type and speed of the charging stations, the state of charge (SoC), battery capacity, duration of charging, and the start time of charging [56]. EV charging stations, also known as EV charging points, provide the essential infrastructure for recharging EV batteries [57]. These stations vary in their power outputs and charging speeds, encompassing different types such as Level 1 charging, which offers basic charging through a standard household outlet with power typically ranging from 1.4 to 2.3 kW; Level 2 charging, which provides faster charging speeds requiring specific charging equipment and delivers 3.3 kW to 22 kW of power; and DC fast charging (Level 3), the fastest charging option, delivering power from 50 kW to 350 kW or more, allowing for rapid recharging of EVs [58–63]. EV charging stations play a crucial role in facilitating the widespread adoption of electric vehicles by providing the necessary infrastructure for battery recharging during travel. Investments by companies, governments, and other entities in the expansion of charging infrastructure are critical to meet the increasing demand for EVs [61].

**Fig 4:**
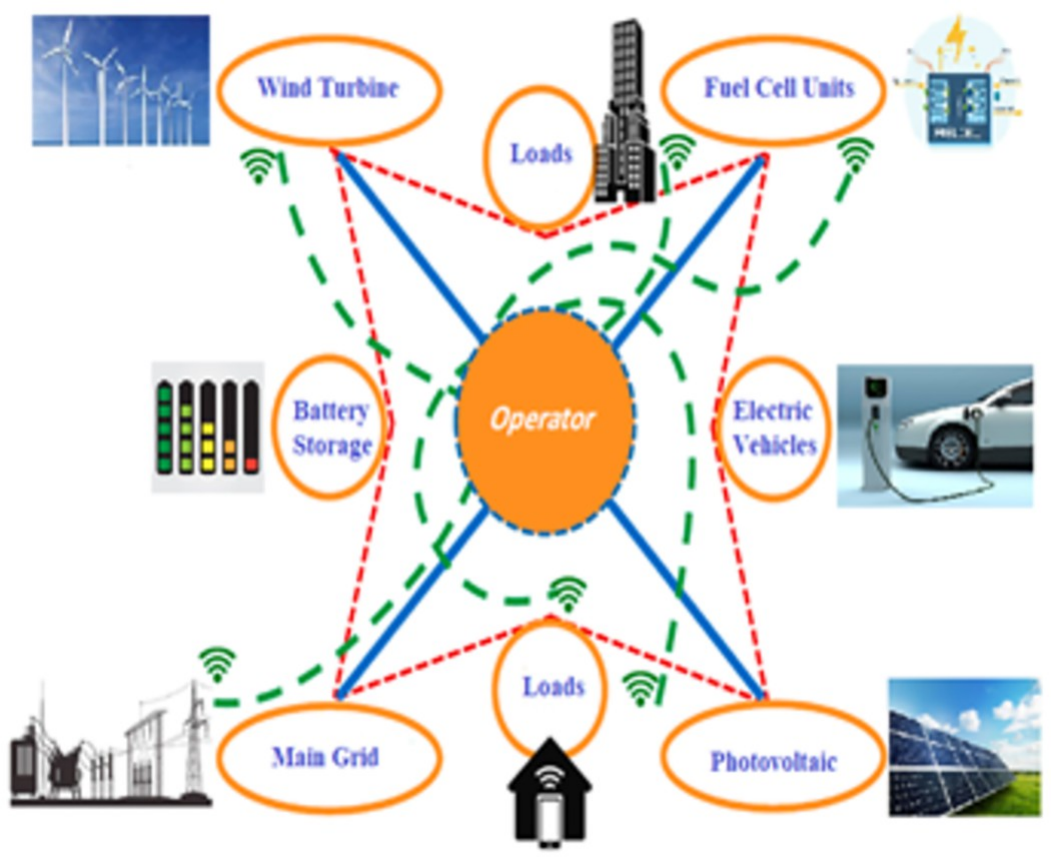
Microgrid configuration.

The integration of EVs into μGs presents both opportunities and challenges. As the adoption of EV grows, there may be an increase in energy demand on the μG. However, EVs can also serve as a flexible load, capable of being managed to match electricity generation and demand profiles within the μG. By charging during periods of high generation and discharging stored energy back to the μG during peak demand or low renewable generation times, EVs can help optimize the use of RES such as solar or wind. Nevertheless, accommodating the rising power demand from EV charging infrastructure, especially from fast-charging stations, may require upgrades to the μG’s distribution system [63]. Thus, the impact of EVs on μGs encompasses both potential benefits and challenges. With appropriate design, smart charging strategies, and integration technologies, EVs can enhance the flexibility, resilience, and sustainability of μGs by managing energy demand, leveraging RESs, and providing distributed energy storage capabilities. A microgrid is defined as a small distribution network equipped with distributed energy resources, BSS, active loads, and EVs, as depicted in Fig 4 [64]. Finally, the μG considered in this study can be described as a compact distribution network that incorporates distributed energy resources, BSS, active loads, and EVs, as depicted in Fig 4 [64].

## 3. Problem formulation

This section outlines the formulation of the optimization problem aimed at identifying the optimal EM strategy for an μG to accomplish either a multi-objective function, which includes minimizing both the total operational cost and emissions, or a single objective function, focusing solely on reducing the total operational cost or emissions. In all three scenarios, the formulation considers various constraints. In a multi-objective optimization model, there are typically several objectives to consider, such as minimizing total operation cost, maximizing the profits of BSS, and minimizing total emissions.

Achieving the optimal balance between these objectives involves finding solutions that offer the best compromise considering assigning relative weights to each objective based on their importance and priorities.

### 3.1 Cost function formulation

This work is dedicated to minimizing the operating costs of an μG, which incorporates PVs, WTs, FCs, BSSs, and EVCSs. The first objective function under consideration (*OF*_1_) is formulated as follows:

$${OF}_{1}=T_{cost}=\text{min}\;f(x_{1})=\sum_{t=1}^{T}\left({MP}_{t}.P_{grid,t}+A_{pv}.P_{pv,t}+B_{wt}.P_{wt,t}+C_{fc}.P_{fc,t}+{CB}_{t}.P_{B,t}\right)$$ (5)

To clarify the provided variables clearly, *MP*_*t*_ represents the market price at time *t* for the utility grid, *P*_*grid*,*t*_ denotes the output power from the main utility at *t*, $A_{pv}$ and *P*_*pv*,*t*_ refer to the bidding cost and the output power of the PVs, respectively. $B_{wt}\;$ and *P*_*wt*,*t*_ indicate the bidding cost and the output power of the WTs. $C_{fc}$ and *P*_*fc*,*t*_ are the cost of the natural gas and the output power of the FC. *CB*_*t*_ and *P*_*B*,*t*_ indicate the bidding cost and the charge or discharge power of the BSS at time *t*.


$${CFC}_{SU}^{t}=\mu_{SU}.max\left(0,\;\left(\alpha_{FC}^{t}-\;\alpha_{FC}^{t-1}\right)\right),\forall \;h$$ (6)

$${CFC}_{SD}^{t}=\mu_{SD}.max\left(0,\;\left(\alpha_{FC}^{t-1}-\;\alpha_{FC}^{t}\right)\right),\;\forall \;h$$ (7)

For the FC, the terms ${CFC}_{SU}^{t},\;{CFC}_{SD}^{t}$, μ_*SU*_, and μ_*SD*_ denote the start-up cost, shut-down cost, and the coefficients for start-up and shut-down costs, respectively. The variable $\alpha_{FC}^{t}$ indicates the operational status of the FC at time *t*, where a value of 1 implies that the FC is active (on), and a value of 0 signifies that the FC is inactive (off).

### 3.2 Emission function formulation

The second objective function (*OF*_2_) is represented as follows:

$$\begin{array}{c} OF_{2}=T_{em}=\text{min}\;f\left(x_{2}\right)=\\ \sum_{t=1}^{T}(\left(\beta_{grid}^{co_{2},t}.\beta_{grid}^{SO_{2},t}.\beta_{grid}^{NO_{2},t}.P_{grid,t}\right)+\left(\beta_{PV}^{co_{2},t}.\beta_{PV}^{SO_{2},t}.\beta_{PV}^{NO_{2},t}.P_{pv,t}\right)\\ +\left(\beta_{WT}^{co_{2},t}.\beta_{WT}^{SO_{2},t}.\beta_{WT}^{NO_{2},t}.P_{wt,t}\right)+\left(\beta_{fc}^{co_{2},t}.\beta_{fc}^{SO_{2},t}.\beta_{fc}^{NO_{2},t}.P_{fc,t}\right)) \end{array}$$ (8)

$\beta_{grid}^{{co}_{2},t},\;\beta_{PV}^{{co}_{2},t},\;\beta_{WT}^{{co}_{2},t}$, and $\beta_{fc}^{{co}_{2},t}$ represent the carbon dioxide emitted from the grid and FC. $\beta_{grid}^{{SO}_{2},t},\;\beta_{PV}^{{SO}_{2},t},\;\beta_{WT}^{{SO}_{2},t}$, and $\beta_{fc}^{{SO}_{2},t}$ represent the sulfur dioxide emitted from the grid and FC. $\beta_{grid}^{{NO}_{2},t},\;\beta_{PV}^{{NO}_{2},t},\;\beta_{WT}^{{NO}_{2},t}$, and $\beta_{fc}^{{NO}_{2},t}$ represent the nitrogen dioxide that is harmful to human health, affecting the respiratory system and is emitted from the grid and FC.

### 3.3 Combined, multi-objective, function formulation

To minimize both the operational costs and pollutant emissions over a 24-hour period, this study introduces multi-objective optimization functions. Below is the formulation of the multi-objective function:

$$OF_{3}=min\;f\left(x_{3}\right)=m_{1}.OF_{1}+m_{2}.OF_{2}$$ (9)

For optimizing both the total operational cost and pollution emissions of the μG over a 24-hour period, *m*_1_ and *m*_2_ represent the weighting factors for the total operational cost and pollution emissions of the μG, respectively. These factors are used to balance the importance of reducing costs versus minimizing environmental impact in the optimization process.

The optimization problem in this paper involves two conflicting objective functions—minimizing operational costs (*OF*_1_) and minimizing environmental impact/pollutant emissions (*OF*_2_). To balance these two objectives, a multi-objective optimization approach can be employed, utilizing weight factors to prioritize the relative importance of each objective. The weight factors, denoted as *m*_1_ and *m*_2_, represent the importance placed on *OF*_1_ and *OF*_2_ respectively. Typically, the range for these weights would be:

$$0\;\leq m_{1}\leq \;1$$ (10)

$$0\;\leq m_{2}\leq \;1$$ (11)

$$m_{1}+m_{2}=1$$ (12)

By adjusting the values of *m*_1_ and *m*_2_, the optimizer can explore the trade-off between the two competing objectives. For example: If *m*_1_ = 1 and *m*_2_ = 0, the optimization would solely focus on minimizing operational costs (*OF*_1_), disregarding environmental impact. If *m*_1_ = 0 and *m*_2_ = 1, the optimization would solely focus on minimizing emissions/environmental impact (*OF*_2_), disregarding operational costs. By using intermediate values, such as *m*_1_ = 0.6 and *m*_2_ = 0.4, the optimization would attempt to find a balanced solution that accounts for both operational costs and environmental impact to some degree. The specific choice of weight factors would depend on the priorities and constraints of the particular μG system being analyzed. For instance, if there are strict emissions regulations or environmental targets to meet, a higher weight (*m*_2_) may be placed on the emissions objective (*OF*_2_). Conversely, if operational costs are the primary concern, a higher weight (*m*_1_) may be placed on the cost objective (*OF*_1_). In this study, the weight factors utilized in the multi-objective optimization were set to *m*_1_ = 0.5 and *m*_2_ = 0.5. This configuration reflected a balanced prioritization between the two competing objective functions: minimizing operational costs (*OF*_1_) and minimizing environmental impact/emissions (*OF*_2_). By assigning equal weights of 0.5 to each objective, the optimization sought to find solutions that provide an equitable compromise between the objectives of reducing operational expenditures and lowering the environmental footprint of the microgrid system.

### 3.4 Constraints considered in the optimization process

The three objective functions are subject to the following constraints:

#### 3.4.1 Energy storage limits

Several constraints related to energy storage should be taken into account in formulating the problem. The first constraint, as presented in Eq (10), relates the charging power $\left(P_{bat-t}^{ch}\right)\;$ to the maximum charging capacity $\left(P_{bat-t}^{ch-max}\right)$ of the BSS. In a similar look, the second constraint, outlined in Eq (11), ensures that the discharged power $\left(P_{bat-t}^{dis}\right)$ does not exceed the BSS’s maximum discharging capacity $\left(P_{bat-t}^{dis-max}\right)$. Eq (15) sets the boundaries for the energy stored within the BSS, adhering to its minimum and maximum *SoC* storage limits.


$$P_{bat-t}^{ch}\;\leq P_{bat-t}^{ch-max},\;\forall t\leq T$$ (13)

$$P_{bat-t}^{dis}\;\leq P_{bat-t}^{dis-max},\;\forall t\leq T$$ (14)

$${SoC}_{t}^{min}\leq {SoC}_{t}\;\leq \;{SoC}_{t}^{max},\;\forall t\leq T$$ (15)

Moreover, the power charged into the battery, accounting for its efficiency (*η*_*bat*_), should match the power discharged, as depicted in Eq (16). The battery’s state of charge at any given time (*SoC*_*t*_) is determined by its previous state (*SOC*_*t*-1_), alongside the quantities of power charged and discharged at time *t*, as specified in Eq (17). It’s important to highlight that for the initial period (*t* = 1), the initial state of charge (*SoC*_0_) must be taken into account. Additionally, the state of charge at the conclusion of the period should revert to *SoC*_0_ to ensure consistency in the battery’s state of charge, as articulated in Eq (18). Eq (19) introduces a constraint related to battery aging, further refining the model’s accuracy.


$$\sum_{t=1}^{T}P_{bat-t}^{dis}=\sum_{t=1}^{T}P_{bat-t}^{ch}.\;\eta_{bat}$$ (16)

$$SoC_{t}=\left\{\begin{array}{ll} SoC_{0}+\;\eta_{bat}\;P_{bat-t}^{ch}\;\Delta t-\;\Delta t\;P_{bat-t}^{dis} & t=1\\ SoC_{t-1}+\;\eta_{bat}\;P_{bat-t}^{ch}\;\Delta t-\;\Delta tP_{bat-t}^{dis} & \forall t\geq 2,\;t\in T \end{array}\right.$$ (17)

$${SoC}_{t}={SoC}_{0},\;t=T$$ (18)

$$B{LC}_{DoD}\geq \sum_{i=1}^{d}\sum_{t=1}^{T}m_{pcb}\left(t,i\right)$$ (19)

#### 3.4.2 Loads balance

At any given time, *t*, the sum of power generated from FCs, PVs, WTs, power imported from (or exported to) the grid, and power discharged from (or charged to) the battery, must balance the total load demand (*P*_*L−t*_) and the EVs’ load (*P*_*EVL*_). This relationship is given in Eq (20).


$$P_{FC-t}i_{t-FC}+P_{PV-t}+\;P_{WT-t}+P_{g-t}+P_{bat-t}=P_{L-t}+P_{EVL},\;\forall t$$ (20)

#### 3.4.3 Power limit of the grid

The power imported from or exported to the grid (*P*_*g−t*_) must adhere to its predefined limits at each time interval, as described in Eq (21).


$$P_{g-t}^{min}\leq P_{g-t}\leq P_{g-t}^{max},\forall t$$ (21)

#### 3.4.4 Power limits of DGs

The output powers of FC, PV, and WT must be within their required limits as expressed in (22)–(24).


$$P_{FC-t}^{min}\;\leq \;P_{FC-t}\;\leq \;P_{FC-t}^{max},\forall t$$ (22)

$$P_{PV-t}^{min}\;\leq \;P_{PV-t}\;\leq \;P_{PV-t}^{max},\forall t$$ (23)

$$P_{WT-t}^{min}\;\leq \;P_{WT-t}\;\leq \;P_{WT-t}^{max},\forall t$$ (24)

### 3.5 Krill Herd Optimization (KHO) employed in this work

Krill Herd Optimization (KHO) is a bio-inspired metaheuristic algorithm modeled after the swarming behavior of krill in the natural world. It has become increasingly popular for its proficiency in addressing complex optimization challenges [65]. In KHO, the objective is often defined by the distance between a krill individual’s position and the target location, representing the search for optimal solutions. The optimization process in KHO comprises three distinct phases: *movement influenced by the presence of other krill*, *foraging behavior*, and *physical random diffusion*. These phases are mathematically formulated to simulate the natural dynamics of krill behavior in seeking optimal positions [66], as follows:

$$\frac{{dX}_{i}}{dt}=M_{j}+V_{j}+P_{j}$$ (25)

where *P*_*j*_ denotes the diffusion of the *j*th krill individuals, *V*_*j*_ denotes the foraging motion, and *M*_*j*_ represents the motion induced by the other krill.

#### 3.5.1 Motion induced by other krill

The initial motion’s (*γ*_*j*_) three main components are the target effect, the local effect, and the repulsive effect. With respect to the krill *j*, the following numerical expression is possible [67].

$$M_{j}^{new}=M^{max}\gamma_{j}+\sigma_{n}M_{j}^{old}$$ (26)

$$\gamma_{j}=\gamma_{j}^{L}+\gamma_{j}^{T}$$ (27)

*M*^*max*^ represents the maximum induced speed, *σ*_*n*_ is the inertia weight within [0,1],${\;M}_{j}^{old}$ denotes the old motion induced, $\gamma_{j}^{L}$ denotes the local effect, $\gamma_{j}^{T}$ denotes the target direction effect.


$$\gamma_{j}^{L}=\sum_{q=1}^{MM}B_{jq}X_{jq}$$ (28)

$$X_{jq}=\frac{X_{q}-X_{j}}{\left\|X_{q}-X_{j}\right\|+\lambda }$$ (29)

$$B_{jq}=\frac{B_{j}-B_{q}}{B_{worst}-B_{best}}$$ (30)

The fitness of the *j*th and *q*th krill, respectively, are *B*_*j*_ and *B*_*q*_. On the krill scale, the best fitness is *B*_*best*_, and the lowest is *B*_*worst*_. *MM* is the total number of neighbors, *B* is the related positions, where *j* = 1,2, …, *MM*.


$$\gamma_{j}^{target}=W_{best}B_{j,best}X_{j,best}$$ (31)

The optimal fitness coefficient for the *j*th krill individual is denoted as *W*_*best*_.

#### 3.5.2 Foraging motion

Two examples of the second action are the location of food and the use of previous experience. The *i*th krill can be expressed as presented below in (32) [68]:

$$F_{j}=V_{f}{£}_{j}+z_{f}F_{j}^{old}$$ (32)

$${£}_{j}={£}_{j}^{food}+{£}_{j}^{best}$$ (33)

$F_{j}^{old}$ is the latest foraging motion, ${£}_{j}^{food}\;$ is the food attractiveness, and ${£}_{j}^{best}$ is the effect of the *i*th krill’s best fitness thus far. *V*_*f*_ is the foraging speed and *z*_*f*_ is the inertia weight in the interval [0,1].

#### 3.5.3 Physical diffusion

**Fig 5:**
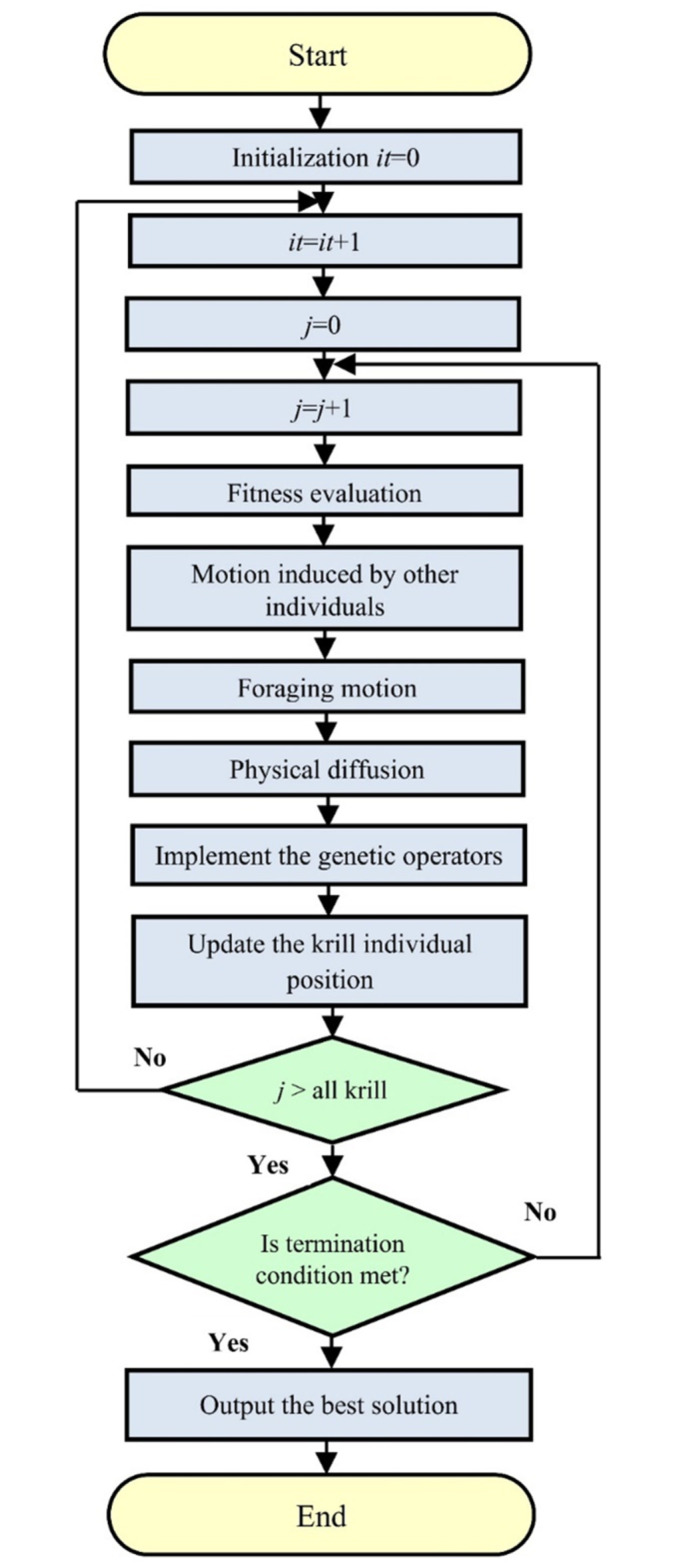
Flowchart of KHO.

In its most basic form, physical diffusion is an unpredictable process [69] expressed as follows:

$$T_{j}=T_{max}\mu$$ (34)

where *T*_*max*_ represents the maximum diffusion speed, and *μ* is a random vector within the range [−1,1]. Drawing inspiration from evolutionary computation, two genetic reproduction mechanisms—namely the crossover and mutation operators—are integrated into the basic KHO algorithm [62]. Detailed descriptions of these genetic reproduction operators and the KHO algorithm are available in [69]. The process is illustrated in the flowchart presented in Fig 5. The steps of how the KHO algorithm is implemented and how nonlinear constraints are considered in the model [70, 71]:

- Initialization: The algorithm begins by initializing a population of potential solutions, referred to as "krill." Each krill represents a candidate solution to the optimization problem.
- Objective Function Evaluation: The objective function, which represents the quantity to be optimized, is evaluated for each krill in the population. The objective function could be defined based on the specific optimization problem, such as minimizing costs or maximizing efficiency.
- Movement and Interaction: The krill in the population move and interact with each other using predefined rules inspired by the behavior of real krill. These rules include attraction to food (good solutions) and repulsion from predators (poor solutions). The movement and interaction aim to explore the search space and improve the solutions iteratively.
- Krill Update: The krill update step involves modifying the positions and characteristics of the krill based on their movement and interaction. The update can be performed using mathematical equations that determine the new positions and characteristics of the krill based on the previous values and the defined rules.
- Constraints Handling: Nonlinear constraints, if present in the optimization problem, need to be considered during the update step. Various approaches can be employed to handle constraints, such as penalty functions, repair mechanisms, or constraint handling techniques like feasibility-based rules. These techniques ensure that the updated solutions satisfy the constraints imposed by the problem.
- Iteration and Termination: The movement, interaction, and update steps are repeated iteratively until a termination condition is met. This condition can be a maximum number of iterations, convergence criteria, or reaching a satisfactory solution based on predefined metrics.

By iteratively updating the positions and characteristics of the krill based on their movement and interaction, the KHO algorithm explores the search space and converges towards optimal solutions. The consideration of nonlinear constraints ensures that the updated solutions satisfy the constraints imposed by the problem, leading to feasible solutions.

## 4. Numerical results and discussion

**Fig 6:**
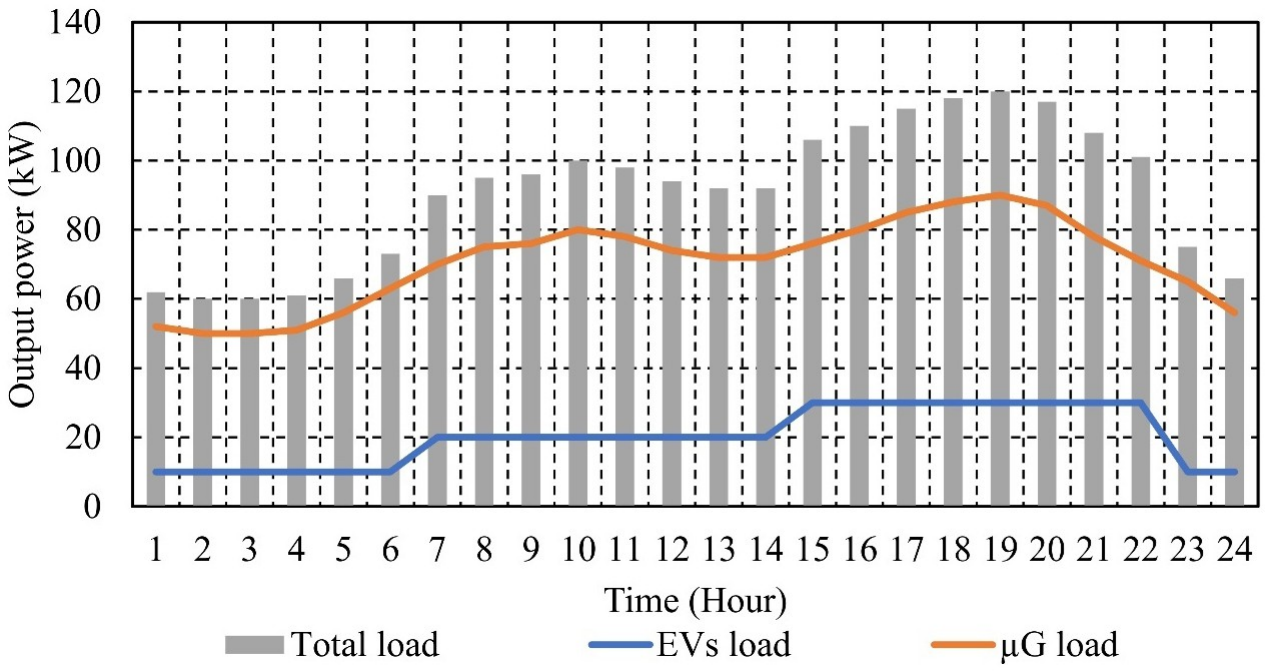
Hourly μG load, EVs load, and total load.

**Fig 7:**
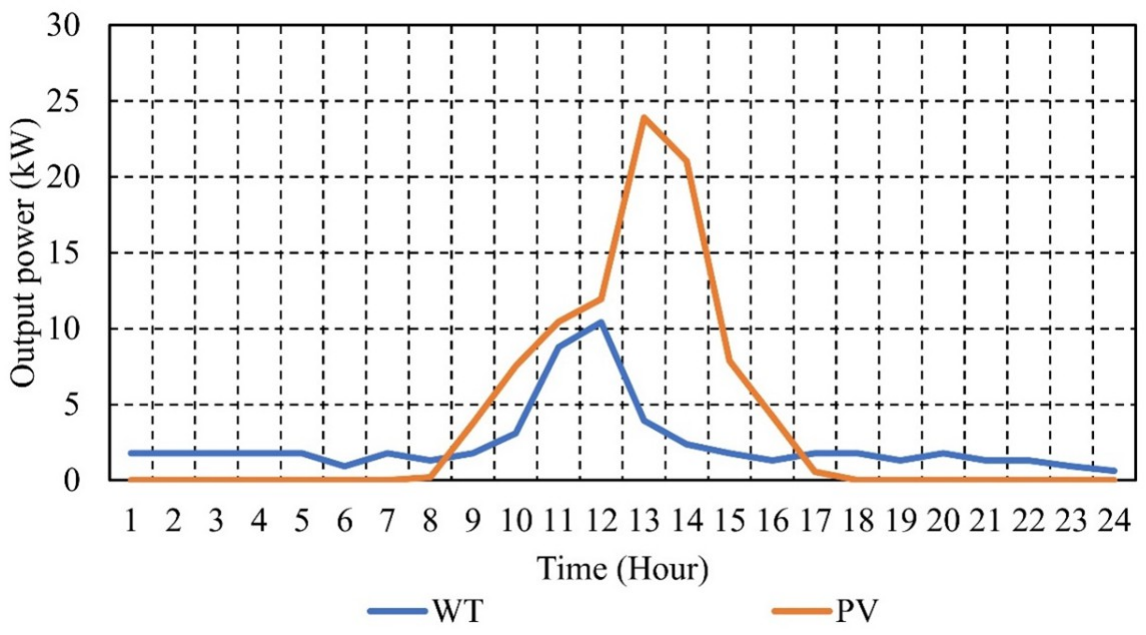
Hourly WT and PV output powers.

This section presents and discusses the results for the optimal EM of the μG under study, employing various objective functions. To evaluate the effectiveness of the proposed EM approach, an hourly changing load pattern was considered, which is depicted in Fig 6. This figure also illustrates the charging profile of the EVs for each hour, enabling the visualization of the total load on the μG, encompassing both the total demand load and the load from EVs, as shown in Fig 6 [72, 73]. Furthermore, Fig 7 displays the hourly projected output power from PVs and WTs for a typical day within the μG outlined in Fig 2 [73].

**Fig 8:**
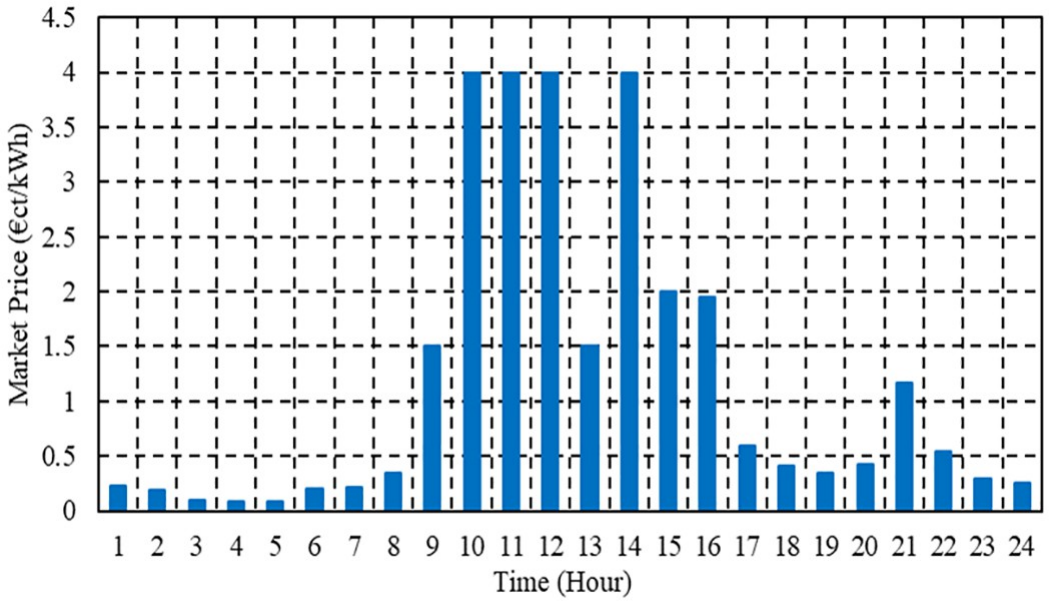
Hourly values of market price during a typical day.

The maximum and minimum power constraints, along with the coefficients used for PVs, WTs, and FCs integrated into the μG, are detailed in Table 1 [74, 75]. Additionally, Fig 8 displays the hourly forecasted energy prices alongside market energy prices during a typical day [74, 75].

**Table 1:** Data of PV, WT, FC, and utilized coefficients.

Parameter	Unit	PV	WT	FC	Grid
Minimum power	kW	0	0	3	0
Maximum power	kW	Fig 7	Fig 7	30	NA
Bid	cent euro /kWh	2.584	1.073	0.294	Fig 5
Start-up/shut-down cost	cent euro	0	0	1.65	0
CO_2_	kg/MWh	0	0	460	720
SO_2_		0	0	0.003	0.0036
NO_x_		0	0	0.0075	0.0075

The current investigation investigates three case studies based on different objective functions:

- Implementing economic dispatch to lower total operational costs and increase BSS profits (Case 1): The objective is to optimize the operation of the μG to minimize operational costs while maximizing the profits generated by the BSS. The objective optimization model would consider factors such as load scheduling, renewable energy generation, energy storage utilization, and grid interaction to find the optimal balance that reduces expenses and maximizes BSS profits.
- Implementing economic dispatch to reduce total emissions from the μG (Case 2): This case focuses on minimizing the environmental impact of the μG by reducing total emissions. The objective would involve optimizing the operation of the μG to prioritize renewable energy generation, minimize reliance on fossil fuels, and maximize energy efficiency. The objective optimization model would aim to identify the operational strategies that result in the lowest emissions while maintaining the microgrid’s constraint.
- Implementing economic dispatch to decrease total operational costs, enhance BSS profits, and cut down total emissions from the μG (Case 3): The objective is to achieve a balanced solution that simultaneously reduces operational costs, increases BSS profits, and decreases total emissions from the μG. The multi-objective optimization model would consider all three objectives and search for solutions that offer the best compromise among them.

The current investigation explores the use of economic dispatch with and without BSS, as well as with various objective functions. Assuming BSS is not implemented, economic dispatch is anticipated to reduce μG’s overall operating cost in Case 1. Second, economic dispatch minimizes the μG’s total emissions in Case 2, either with or without BSS. To optimize BSS revenues and overall μG emissions while minimizing total operating cost, economic dispatch is used in Case 3.

**Fig 9:**
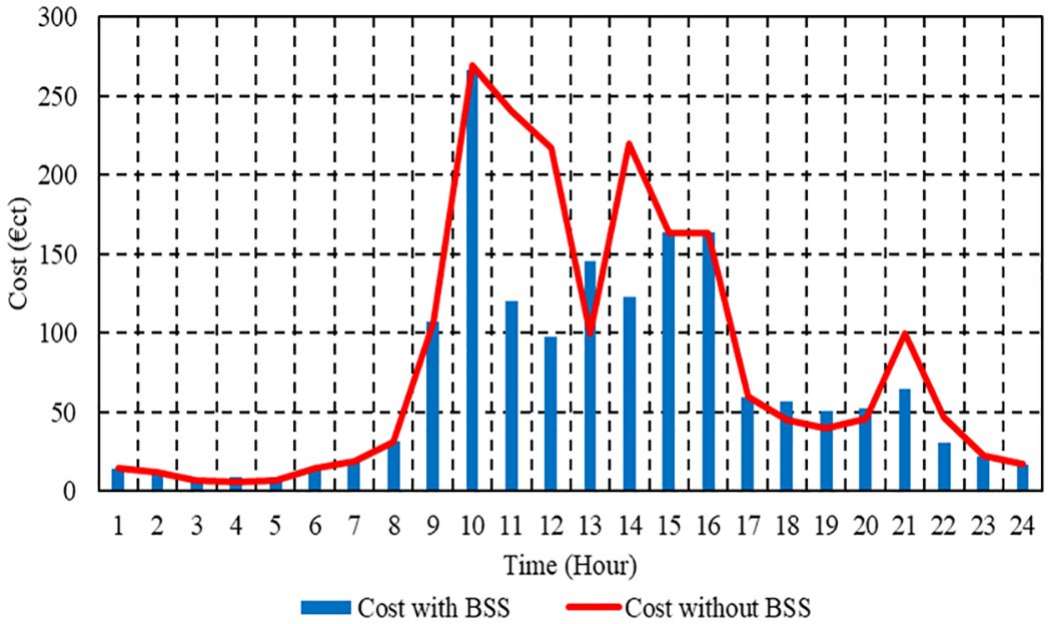
Hourly operation cost of the μG with and without BSS in Case 1.

In Case 1, the total operation cost is depicted in Fig 9 for each hour with and without BSS. Using BSS results in a daily savings of 15.3% compared to μG’s overall operation cost of 1970.58 cent euros when operating without ESS. The total operation cost of the μG with BSS is 1659.83 cent euros. Operating expenses with BSS are reduced for μG compared to those without BSS, which is apparent clearly in Fig 9.

**Fig 10:**
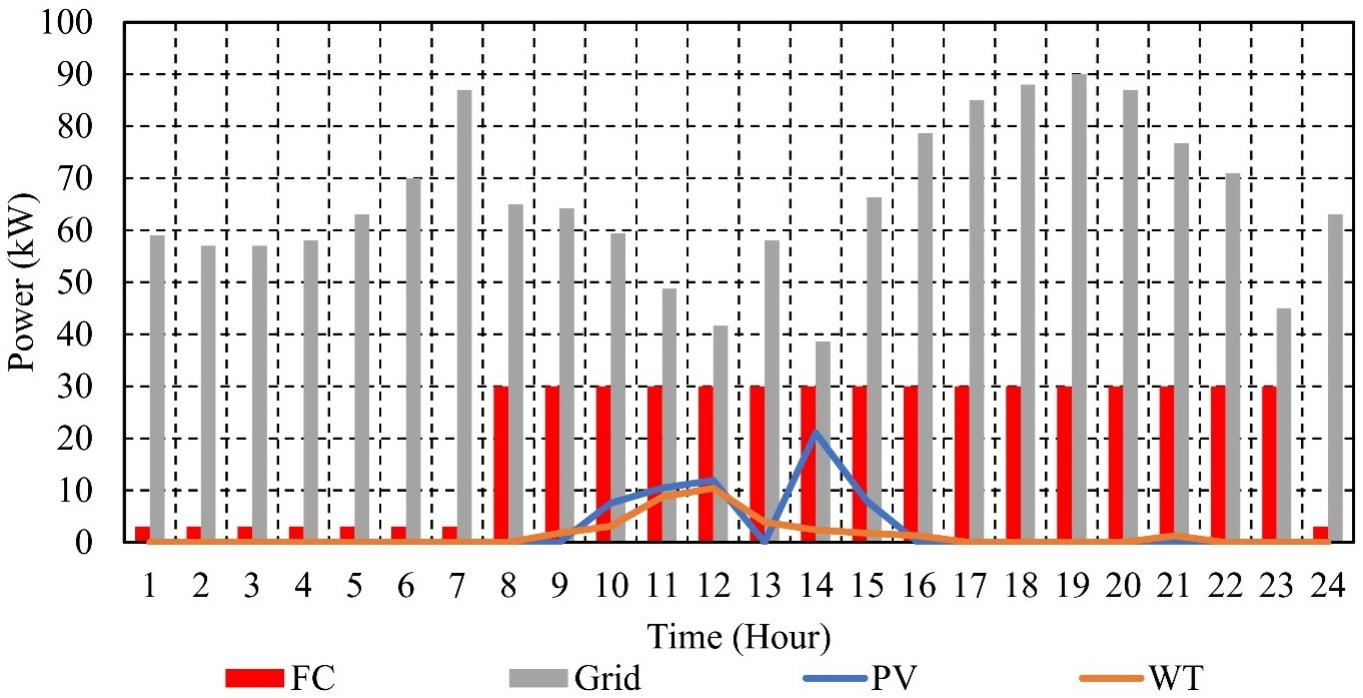
EM of the μG with no BSS in Case 1.

**Fig 11:**
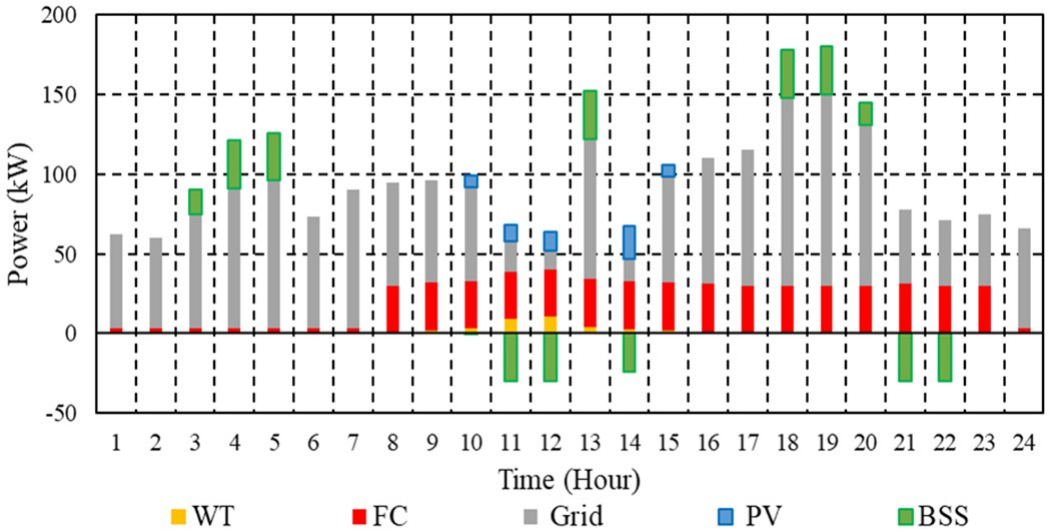
EM of the μG with BSS in Case 1.

To minimize operating cost without BSS, the EM of the suggested μG is presented in Fig 10. Fig 11 depicts the EM of the μG that was investigated in order to maximize BSS profitability while minimizing operating costs. The emissions of CO2 from the μG with BSS amount to 1287.9 kg, whereas those from the μG without BSS amount to 1392.1 kg. The daily decrease in emissions caused by the use of ESS is 7.5%. Using BSS reduces SO₂ emissions by 6.2% daily, with a total of 0.00686 kg emitted by the μG and 0.00731 kg emitted by the μG without BSS. The overall NOx emissions from the μG with BSS are 0.01519 kg, whereas those from the μG without BSS are 0.015865 kg; the daily decrease in emissions is 4.3% as a result of adopting BSS. The results indicate that making use of BSSs reduces the emission of the μG.

**Fig 12:**
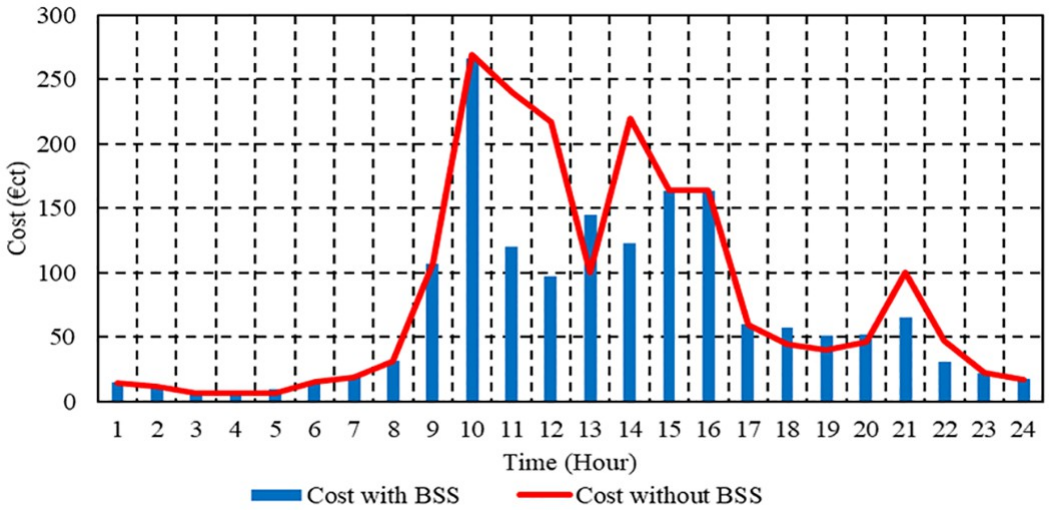
Hourly operation cost of the μG with and without BSS in Case 2.

Case 2’ s total operation cost per hour with and without BSS is illustrated in Fig 12. Using BSS results in a daily savings of 15.1% compared to the overall operating cost of the μG without BSS, which is 2048.9 cent euros. Operating expenses with BSS are lower for μG than those without it, as shown clearly in Fig 12.

**Fig 13:**
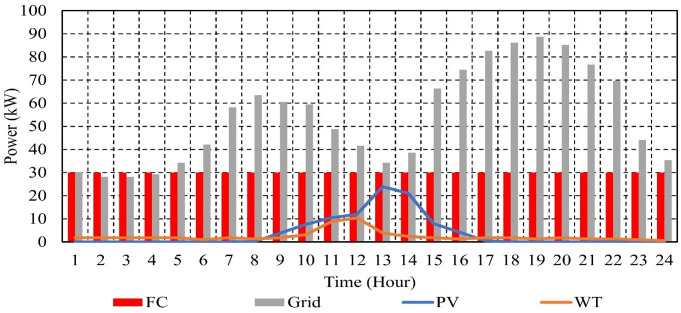
EM of the μG with no BSS in Case 2.

Fig 13 depicts the EM strategy for the μG under consideration in order to minimize operational costs in the absence of BSS.

**Fig 14:**
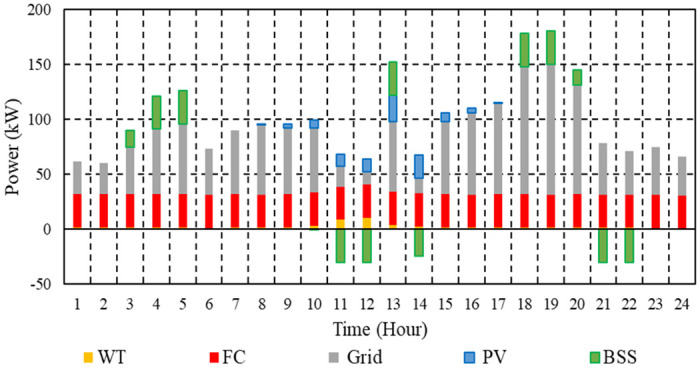
EM of the μG with BSS in Case 2.

Fig 14 displays the EM strategy for the μG under consideration, which aims to maximize BSS earnings while minimizing operating costs. Every day, making use of BSS reduces the overall CO_2_ emissions by 2.1% for μGs equipped with BSS, which comes to 1260.7 kg, compared to 1287.9 kg for μG without BSS.

The total SO₂ emissions from the μG with BSS are 0.00678 kg, whereas those from the μG without BSS are 0.00686 kg. The daily percentage decrease is 1.1% as a result of utilizing BSS. A daily decrease of 1.2% is attributable to the use of ESS, as the total NOx emissions from μGs equipped with BSS are 0.015 kg, while those from μGs without BSS are 0.01519 kg. The results demonstrate that using BSS reduces the emissions of the μG.

The penetration level of renewable energy sources (PV and WT) within the μG under consideration is relatively low compared to the total system load. The proportion of energy supplied by renewable generation assets is small in relation to the aggregate power consumption across the μG. Therefore, the difference between Figs 9 and 12 is simple.

At hour 13, the total power generated by PV and WT exhibits a notable increase relative to other hours within the day. Consequently, this hour presents a distinct cost disparity between Case 1 and Case 2. The operational cost of the μG in Case 1, incorporating BSS, amounts to 145.1 cent euros, while the operational cost of the μG in Case 2 with BSS stands at 171.1 cent euros, resulting in a difference of 26 cent euros in the day shown.

**Fig 15:**
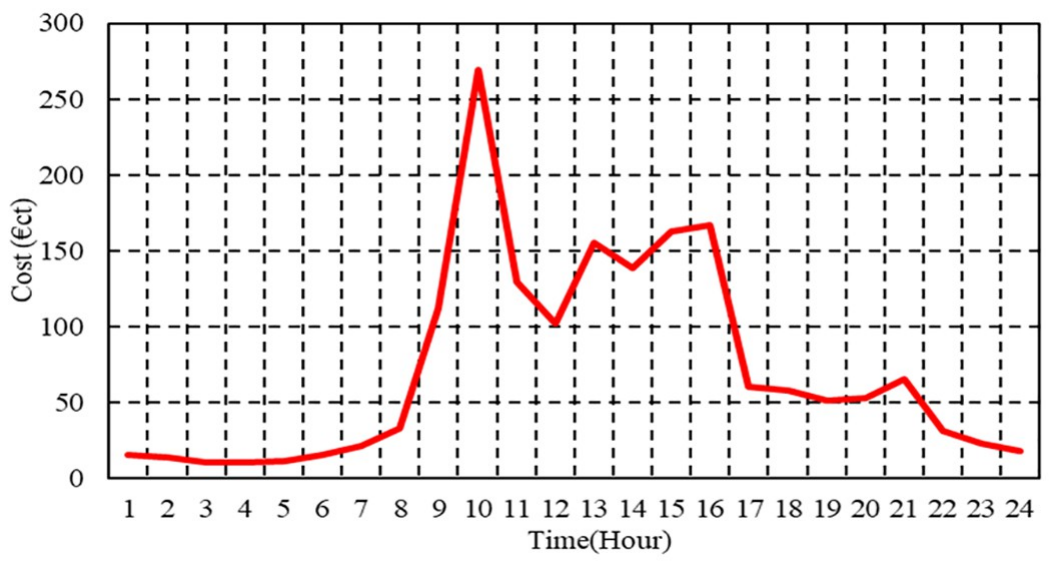
Hourly operation cost of the μG with BSS in Case 3.

**Fig 16:**
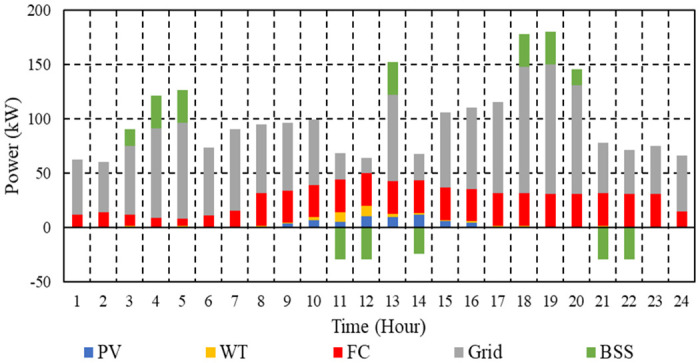
EM of the μG with BSS in Case 3.

The overall cost of operation per hour with BSS in Case 3 is shown in Fig 15. With ESS, the total operating cost of the μG is 1,732.3 cent euros. To minimize total emissions, maximize total revenues of BSS, and minimize operating costs, the energy administration of the investigated μG is depicted in Fig 16.

The daily operating costs of the μG with BSS in Cases 1, 2, and 3 are 1659.83 cent euros, 1738.16 cents, and 1723.8 cent euros, respectively. For Case 1, the total CO_2_ emissions from the μG with BSS per day are 1287.9 kg; for Case 2, they are 1260.7 kg; and for Case 3, they are 1278.2 kg. In Case 1, the total daily SO₂ emissions from the μG with BSS amount to 0.00686 kg; in Case 2, they amount to 0.00678 kg; and in Case 3, they amount to 0.00683 kg. Also, the daily NOx emissions from the μG with BSS in the first Case are 0.01519 kg; in Case 2, they are 0.015 kg; and in Case 3, they are 0.01509 kg. The results show that Case 1 with BSS has the best cost, case 2 with BSS has the best emission, and Case 3 with BSS has moderate operation cost and emissions.

The KHA is used to solve the considered optimization problem. The validity and effectiveness of the proposed solution are demonstrated by comparing the results obtained by other optimization methods such as the FF, CK, and GSS algorithms.

Table 2 presents the results of the total operating cost of the μG (cent euros) and Total CO_2_, SO_2_, and NO_x_ emissions by using different optimization techniques such as KHO, FF, CK, and GAS algorithms in different cases. It is clear from the results presented in Table 2 that the KHA provides the lowest total operating cost of the μG and Total CO_2_, SO_2_, and NO_x_ emissions compared to the results obtained by the other optimization algorithms in the different cases.

**Table 2:** The obtained outcomes utilizing various optimizers in various cases.

Optimizers		KHO	FF	CK	GSS
Case 1 with BSS	The total operating cost of the μG (cent euros)	1659.83	1665.6	1673.6	1685.9
	Total CO_2_ emissions (kg)	1287.9	1296.6	1302.8	1315.7
	Total SO₂ emissions (kg)	0.00686	0.00691	0.00695	0.00701
	Total NOx emissions (kg)	0.01519	0.01602	0.01681	0.01725
Case 2 with BSS	The total operating cost of the μG (cent euros)	1738.16	1744.6	1752.6	1779.6
	Total CO2 emissions (kg)	1260.7	1268.9	1271.5	1298.9
	Total SO₂ emissions (kg)	0.00678	0.00692	0.00712	0.00732
	Total NOx emissions (kg)	0.015	0.0161	0.0169	0.0256
Case 3 with BSS	The total operating cost of the μG (cent euros)	1723.8	1731.2	1738.2	1743.2
	Total CO2 emissions (kg)	1278.2	1290.2	1298.2	1213.6
	Total SO₂ emissions (kg)	0.00683	0.00715	0.00795	0.00805
	Total NOx emissions (kg)	0.01509	0.01621	0.01702	0.01796

## 5. Conclusions

This paper presents a comprehensive optimization framework for reducing operational costs and emissions in μGs. The framework considers the loads from EV charging stations and integrates BSS to optimize the energy management of interconnected μGs. The proposed day-ahead scheduling model incorporates various energy sources such as PVs, FCs, and WTs, along with BSS and EV charging stations. The study demonstrates the effectiveness and benefits of coupling BSS with EV charging stations in the context of μGs. The framework offers insights into cost and emission reductions, highlighting the potential for enhancing energy efficiency and sustainability. The results showcase the performance of the optimization model and its ability to achieve optimal μG operations. This research contributes to the field by providing a novel approach to address the operational challenges of μGs, paving the way for more efficient and environmentally friendly energy management strategies. The key insights from this investigation can be summarized as follows:

- Analysis of three distinct objective functions revealed that the operational costs for the μG equipped with BSS are consistently lower compared to configurations lacking ESS.
- Similarly, the emission levels from the μG with BSS implementation are significantly reduced in comparison to scenarios without ESS.
- Focusing on minimizing μG’s operational costs, the study found that the total costs with ESS amounted to 1659.83 cent euros, whereas prioritizing emission reduction shifted the total operational costs to 1738.2 cent euros, achieving a daily savings of 4.5%.
- When the objective was cost minimization, the CO_2_ emissions from the μG with ESS stood at 1392.1 kg. However, targeting emission reductions directly, the emissions dropped to 1260.7 kg, marking a 9.4% daily savings.
- Under a multi-objective optimization approach, the operational cost of the μG with ESS was calculated at 1332.4 cent euros, with CO_2_, SO_2_, and NO_2_ emissions measured at 1332.4 kg, 0.007 kg, and 0.01538 kg, respectively.
- The results indicate that the most balanced approach in terms of μG operational cost and emissions operating costs and emissions.
- In every instance, KHA outperformed other optimization methods including FF, CK, and GAS in terms of performance.
- Performance was achieved in Case 3, which simultaneously targets both the minimization of These findings underscore the potential of integrating BSS with EV charging stations in μGs, not only for enhancing energy efficiency and sustainability but also for achieving significant reductions in both operational costs and environmental impact.

Building upon these findings, future research could focus on expanding the scalability of these systems to include more diverse renewable energy sources. Further exploration into advanced BSS control strategies using artificial intelligence (AI) or machine learning (ML) could optimize energy storage and distribution while considering demand response and customer satisfaction scenarios. Additionally, assessing the impacts of higher EV penetration rates on grid stability and infrastructure needs will be crucial to ensure their contribution to sustainable energy goals. The progression towards smart grids necessitates ongoing innovation and research in these areas to fully realize the potential of renewable energy and EVs in contributing to a sustainable energy future.
